# Circ_001209 aggravates diabetic retinal vascular dysfunction through regulating miR-15b-5p/COL12A1

**DOI:** 10.1186/s12967-021-02949-5

**Published:** 2021-07-07

**Authors:** Fang Wang, Meixia Zhang

**Affiliations:** 1 Department of Ophthalmology, West China Hospital, Sichuan University, Chengdu, 610041 Sichuan China; 2 Department of Ophthalmology, The Affiliated Hospital of Southwest Medical University, Luzhou, China

**Keywords:** circ_001209, Diabetic retinal, HRVECs, miR-15b-5p, COL12A1

## Abstract

**Objective:**

Diabetic retinopathy, a common complication of diabetes mellitus and a major cause of blindness. circRNAs spongs target miRNA and thus influencing mRNA expression in DR. We investigated the mechanism of circ_001209 in regulating diabetic retinal vascular dysfunction.

**Methods:**

QRT-PCR analysis was performed to detect the expression of miR-15b-5p, COL12A1 and circ_001209 in human retinal vascular endothelial cells (HRVECs) under high glucose conditions. Western blot assay, wound healing assay, transwell assay and tube formation were used to explore the roles of circ_001209/miR-15b-5p/COL12A1 in retinal vascular dysfunction. Bioinformatics analysis and luciferase reporter, RNA-FISH, and overexpression assays were performed to reveal the mechanisms of the circ_001209/miR-15b-5p/COL12A1 interaction. TUNEL staining and H&E staining were used to evaluate the pathological changes in streptozotocin (STZ)-induced DR in rats.

**Results:**

Downregulation of miR-15b-5p under HG conditions promoted proliferation, migration, and tube formation of HRVECs. QRT-PCR and western blot results revealed that miR-15b-5p affected the HRVECs function through targeting COL12A1. Under HG conditions, circ_001209, which acts as a sponge of miR-15b-5p, is upregulated. Besides, overexpression of circ_001209 can affect HRVEC function and aggravate retinal injury in diabetic rats.

**Conclusion:**

Upregulation of circ_001209 contributes to vascular dysfunction in diabetic retinas through regulating miR-15b-5p and COL12A1, providing a potential treatment strategy for diabetic retinopathy.

## Introduction

Diabetic retinopathy (DR), as one of the most common complications of diabetes mellitus (DM), which is the leading cause of vision impairment and even blindness worldwide [1]. The disease reportedly affects more than 100 million patients worldwide as of 2010 and will continue to rise, reaching an estimated 190 million by 2030 [2]. Previous studies [3] have reported that diabetic retinas are mainly accompanied by microaneurysms, punctate hemorrhage, cotton-floss plaques and retinal microvascular abnormalities under continuous hyperglycemia stimulation. The pathological manifestation of diabetic retinopathy is that the patient's continuous hyperglycemia environment leads to the apoptosis of retinal peripheral vascular cells, promotes the abnormal proliferation of retinal vascular endothelial cells, and seriously affects the function of retinal blood vessels [4–6]. At present, the primary treatment strategy [7] is still to control the body’s blood glucose concentration and alleviate the sustained damage caused by hyperglycemia stimulation, but the mechanism of diabetic retinopathy targeting vascular endothelial function is still unclear. Thereby, further research on the mechanism of DR progression is urgently needed to provide a new effective therapeutic strategy for DR.

MicroRNAs (miRNAs) are small, non-coding RNAs that interact with the 3’ untranslated region (UTR) of mRNAs to directly regulate mRNA degradation and translation, and regulates gene expression. It has been reported that miRNAs have a wide range of regulatory functions, including cell proliferation, differentiation, apoptosis, autophagy and other physiological and pathological processes, which may indirectly affect physiological functions and pathological processes of the body [8]. Increasing evidences [9] have showed that miRNAs are associated with the pathological process of DR. Xue et al. [10] found that miR-200-3p plays a protective role in inhibiting the proliferation of diabetic retinopathy cells by blocking the TGF-2/Smad pathway. Ji et al. [11] suggested that miR-34a promotes apoptosis of RVECs by targeting SIRT1 in DR rats, which provide a valuable target for DR treatment. In Ye et al. [12] reported, miR-15b plays a protective role by regulating the TNF-α/SOCS3 signaling pathway against hyperglycemia-induced retinal endothelial cell apoptosis.

CircRNA, as a novel star in the study of ncRNA, has a single-chain covalent closed structure, excluding 50 caps and 30 multi-A-tails, and its mechanism of action in the field of biomedicine has attracted wide attention [13]. With the rapid development of biological technologies, circRNAs have been found to interact with RNA-binng proteins (RBPs) to regulate RNA splicing or transcription and translation into peptides or proteins to regulate various biological processes, including cell differentiation, proliferation, apoptosis, inflammation and invasion, in addition to acting as sponges for microRNAs [14]. It is reported that circ_0005015 may serve as a novel potential biomarker of DR, which could facilitate retinal endothelial angiogenic function and aggravate the progression of DR. Combined with the database to predict the upstream circRNA of miR-15b-5p, we further explored the regulatory mechanism of circ_001209 in diabetic retinopathy.

In our study, we analyzed the expression of miR-15b-5p and circ_001209 in the HRVECs induced by HG and diabetic rats. We found that overexpression of circ_001209 could promote the cell proliferation, migration and vascularization and in HG-induced HRVECs. Besides, we also confirmed that circ_001209 could aggravate the progression of DR in vivo through interaction with miR-15b-5p/COL12A1.

## Materials and methods

### Cell culture and transfection

Human retinal vascular endothelial cells (HRVECs) were purchased from Cell Systems Corporation (UK). HRVECs were cultured in a basic endothelial cell culture medium (5 mM glucose) containing 10% FBS (fetal bovine serum) in an incubator at 37 °C with 5% CO_2_, called the control group. In the HG group, the cells were treated with 25 mM glucose (Solarbio, Beijing). When the HRVECs coverage reached 80% of the culture flask, cell passage was started, and cells at passage three to eight were selected for subsequent experiments. HRVECs were transfected with miR-15b-5p mimics, mimics control, pcDNA3.1 (empty plasmid) and pcDNA3.1-COL12A1 (RiboBio, China) by Lipofectamine 3000 (Thermo Fisher Scientific, USA) according to the manufacturer’s instructions. The relative expressions of miR-15b-5p and COL12A1 were detected by qRT-PCR analysis at 48 h after transfection.

### Diabetic retinopathy model

The study was approved by the Animal Ethics Committee of West China Hospital, and all experiments are conducted in accordance with guidelines for the use and care of laboratory animals, following the principles of reducing animal numbers and suffering. A total of 80 Sprague–Dawley male rats (160 ± 10 g) were purchased from Beijing huafukang bioscience co., LTD. Rats were kept in ventilated cages in controlled room (12 h light–dark cycle) with a temperature of 25 ± 1 °C and humidity of 50%. Under the conditions of the environment permits, the rats were allowed to free access to water and food. After seven days of adaptive feeding, animal experiments were started. Diabetic retinopathy rats were induced by intraperitoneal injection of 65 mg/kg STZ (streptozotocin, Sigma, USA) [15]. The rats were fasted for 12 h before injection. Then, STZ was dissolved in 10 mM citrate buffer (pH = 4.5) and injected continuously for 7 days to induce DR model. At the same time, the sham rats were intraperitoneally injected with sodium citrate buffer. After the last injection, blood was collected from the tail vein for blood glucose detection. A blood glucose meter was used to detect blood glucose concentration > 16.7 mmol/L for consecutive seven days, indicating that the diabetes model was successful. Besides, fasting blood glucose was monitored and recorded every other week after the end of injection. Adequate food and water were given during the feeding period and keep the cage clean every day.

### Intravitreal injection of AAV-circ_001209

The right eye of the rats was given topical treatments with 1% atropine sulfate and 2.5% phenylepinephrine hydrochloride to expand the eyes, making the injection more convenient. The human circ_001209 sequence was cloned into AAV2 vector and packaged into adeno-associated virus (AAVs) by General Biotech. Four μL AAV-circ_001209 or AAV-ciR-NC (empty vector) (7 × 10^13^ vg/mL) was injected into the vitreous cavity of rats using a microinjector. The relative expression level of circ_001209 in the retinal tissue was detected by qRT-PCR analysis to evaluate the transfection efficiency.

### Quantitative real-time polymerase chain reaction (qRT-PCR) analysis

Total RNA was extracted from retinal tissue and HRVECs using the TRIzol reagent (Invitrogen, USA) according to the protocols. The RNA was reverse-transcribed into cDNA using the PrimeScriptTM RT Reagent Kit (TaKaRa, Japan). SYBR Premix Ex TaqTM was used for amplification. GAPDH was used as an internal control for mRNA and circRNA, while U6 was selected as an internal control for miRNAs. The relative mRNA expressions in HRVECs and tissues were calculated using 2^−ΔΔCt^ method.

### Western blotting assay

Western blotting assay was used to detect the protein expression of COL12A1 and GAPDH in HRVECs. Firstly, proteins were extracted from the cells using RIPA lysis buffer. Protein concentration in the samples was determined by BCA kit and diluted to 10 μg/μL with PBS. Then, the samples were separated by 10% sodium dodecyl sulfate polyacrylamide gel electrophoresis and transferred to PVDF membrane (Millipore, USA). The membrane was blocked with 5% skim milk at room temperature for 3 h, and the primary antibody (COL12A1 and GAPDH) was incubated overnight at 4 °C. After washing the membrane for three times with TBST, the secondary antibody was incubated at 37 °C for 1 h. ECL-chemiluminescent kit (Thermo Scientific, USA) was used to develop and quantify protein expression levels. Finally Image J was used to analyze the gray values of the protein bands, and GAPDH was used as the loading control.

### Wound healing assay

Wound healing assay was used to detect the migration of HRVECs. As previously reported, the HRVECs were seeded in 24-well plate, and the cell confluency reached over 90%, the scratch was made by a pipette tip. Then the cells were cultured for 24 h, and the scratches were observed with a microscope (Leica, Germany) to evaluate the cell migration ability.

### Transwell assay

Transwell assay was used to detect the cell migration and invasion. Briefly [16], the cells were seeded on the top layer of transwell chambers (Corning, USA) in serum-free medium, and then allowed to migrate to the lower compartment contained complete media with 10% FBS in an incubator at 37 °C for 24 h. The migrated cells were fixed with fixative solution for 10 min and stained with 0.1% crystal violet for 15 min. After washing with PBS, the cells were photographed and quantified with Image J software.

### Tube formation assay

To investigate the ability of cell angiogenesis, tube formation assay was performed in vitro [17]. Growth Factor Reduced Matrigel matrix was evenly dispersed in 24-well plates and incubated at 37 °C for 1 h. The cells were seeded on the matrix surface at a density of 1 × 10^5^ cells/well and cultured in an incubator for 24 h. Finally, the capillary-like structures were observed under Nikon inverted microscope and its branches were analyzed by image J software.

### Dual luciferase reporter assay

According to the database prediction, we designed primer sequences, and vectors containing relevant primer sequences were synthesized by general biosynthesis for subsequent experiments. 293T cells were seeded in 24-well plates (1 × 10^5^ cell/well) and co-transfected with circ_001209/COL12A1 wt or mut plasmid and miR-15b-5p mimics or mimics control using Lipofectamine 3000 (Thermo Fisher Scientific, USA). At 48 h after transfection, luciferase activity was measured by the dual luciferase reporter assay system (Promega) following to the protocol [18].

### RNA fluorescence in situ hybridization (FISH)

RNA FISH assay was detected using fluorescent In Situ Hybridization kit (RiboBio, China) following the manufacturer’s protocol. The specific probes targeting circ_001209 and miR-15b-5p were used in the FISH experiment. In brief [19], HRVECs were fixed in 4% polyformaldehyde at room temperature for 15 min and washed twice with PBS. Subsequently, the penetration treatment was performed with 0.5% Triton. Then cells were hybridized overnight at 37 °C in darkness and humidity. The next day, the cells were washed with sodium citrate buffer and incubated in the blocking solution for 1 h at room temperature. The HRVECs were incubated with HRP-conjugated anti-biotin antibody at 4 °C overnight. Finally, images were observed and taken using a confocal microscope (Leica, Germany).

### Terminal deoxynucleotidyl transferase (TdT)-mediated dUTP biotin nick end labeling (TUNEL) staining

Briefly, the retina tissues were embedded in paraffin embedded were made into 5 μm-thick sections, extended in warm water at 42 °C, mounted, and baked. Subsequently, the resulting sections were added drop wise with TdT reaction solution and reacted in the dark for 1 h. Then, the reaction was terminated through incubation with deionized water in drops for 15 min. After the activity of endogenous peroxidase was blocked by hydrogen peroxide, the sections were added drop wise with working solution. Following reaction for 1 h, the sections were rinsed, added with DAB solution in drops for color development, being counter-stained with hematoxylin for 20–30 s, and washed in running water again. Finally, they were dehydrated in increasing concentrations of alcohol sealed and observed. The apoptotic index was determined by counting the percentage of positive cells in 6 randomly chosen fields.

### Hematoxylin–eosin (H&E) staining

Retinal tissue was carefully separated from rats, gently washed with 1% PBS (phosphate buffered saline), and fixed with 4% paraformaldehyde for 2 h for subsequent experiments. The tissue was embedded in paraffin and cut uniformly into 5-μm thick sections. As described previously [15], sections were used for H&E staining to observe pathological changes in DR rats. After the staining, it was naturally air-dried, observed under a light microscope and photographed.

### Statistical analysis

The in vitro experiments in the study were performed at least three independent replicates and the data was presented as mean ± SEM. SPSS 22.0 software was used to analyze the data. The differences between two groups were analyzed by unpaired Student’s *t* test. Multiple comparisons were calculated using one-way analysis of variance (ANOVA), followed by Bonferroni multiple comparison test. P < 0.05 was considered statistically significant.

## Results

### MiR-15b-5p is low expressed in STZ-induced diabetic retinopathy tissues and affects function of HRVECs under high-glucose (HG) conditions.

**Fig. 1:**
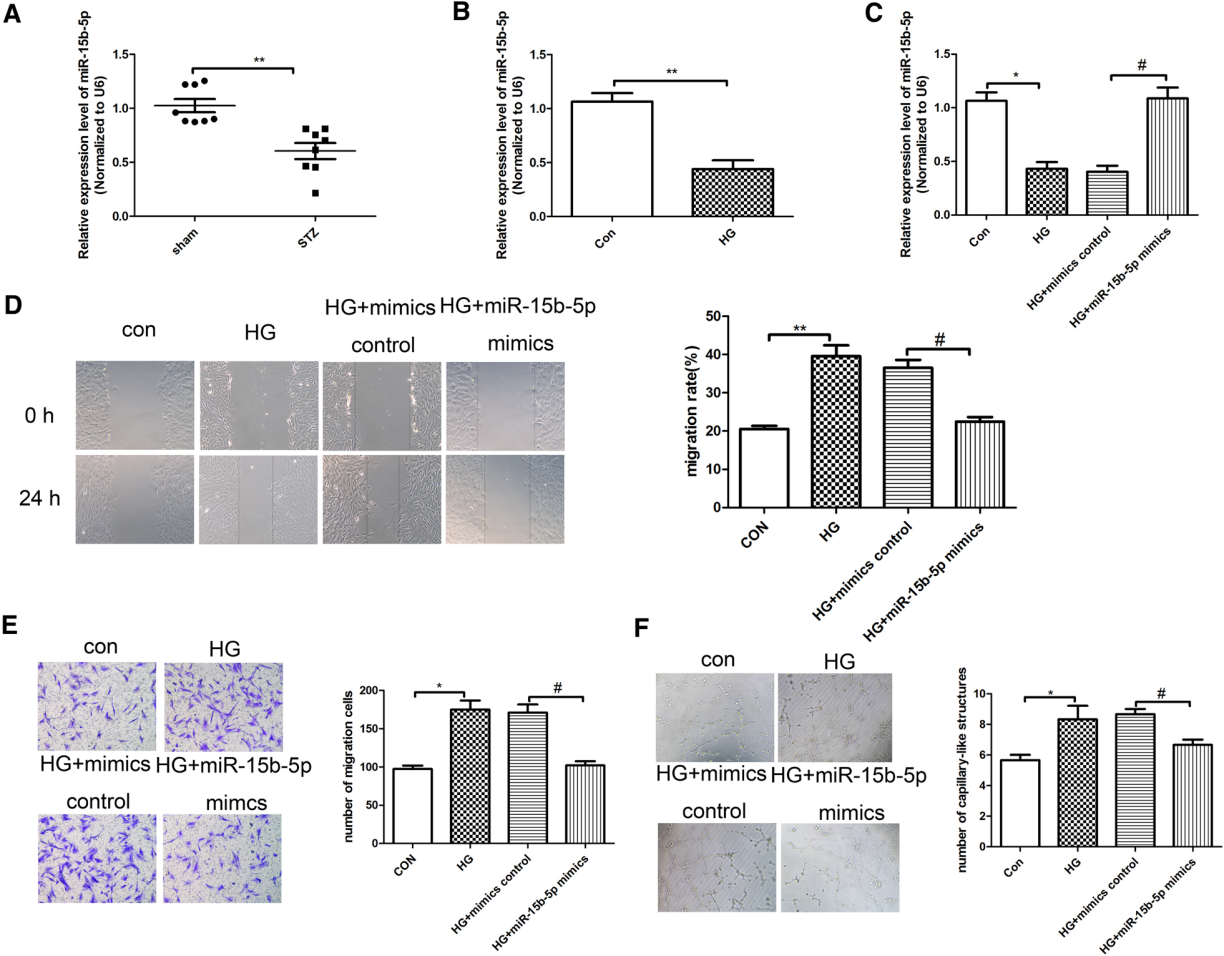
MiR-15b-5p is low expressed in STZ-induced diabetic retinopathy tissues and affects function of HRVECs under high-glucose (HG) conditions. **A** The relative expression levels of miR-15b-5p, detected by qRT-PCR analysis, in sham group and diabetic rat retinas. **B** The qRT-PCR analysis was used to detect the expression level of miR-15b-5p in HRVECs induced by HG (25 mM glucose). **C** The relative expression of miR-15b-5p in HRVECs was detected by qRT-PCR analysis. **D** Wounding healing assay was performed to detect the invasion ability in different groups. **E** Transwell assay was used to measure the migration ability in different groups. **F** The tubule-forming ability of cells was measured by the tubule formation experiment. The data were presented as mean ± SEM, n = 3 each group, vs con/sham group, *P < 0.05, **P < 0.01; vs HG + mimics control group, ^#^P < 0.05

We detected the expression levels of miR-15b-5p in STZ-induced diabetic retinopathy tissues and HRVECs induced by HG. As shown in Fig. 1A, compared with sham group, the expressions of miR-15b-5p was reduced significantly in STZ-induced diabetic retinopathy tissue. We cultured the HRVECs in high-glucose (HG) (25 mM) and control group (5 mM) and followed by qRT-PCR analysis. The results (Fig. 1B) showed that compared with control group, the expression of miR-15b-5p was down-regulated markedly in HG group. To further investigate the role of miR-15b-5p in diabetic retinopathy, HRVECs were transfected with mimics control and miR-15b-5p mimics and cultured in HG and control conditions, respectively. The expression of miR-15b-5p was measured by qRT-PCR analysis in HRVECs in different groups. The results (Fig. 1C) revealed that compared with HG + mimics control group, the expression of miR-15b-5p was increased significantly in HG + miR-15b-5p mimics group. Furthermore, we detected the invasion, migration, and angiogenesis of HRVECs induced by HG through wound healing assay, transwell assay and tube formation experiment. As shown in Fig. 1D–F, it showed that HG significantly increased the invasion, migration and tubular formation of HRVECs cells. Compared with HG + mimics control group, the effects of HG were reversed with miR + 15b-5p mimics. Taken together, we found that miR-15b-5p could significantly inhibit invasion, migration and tubular formation induced by HG.

### MiR-15b-5p regulates HRVEC function through targeting COL12A1

**Fig. 2:**
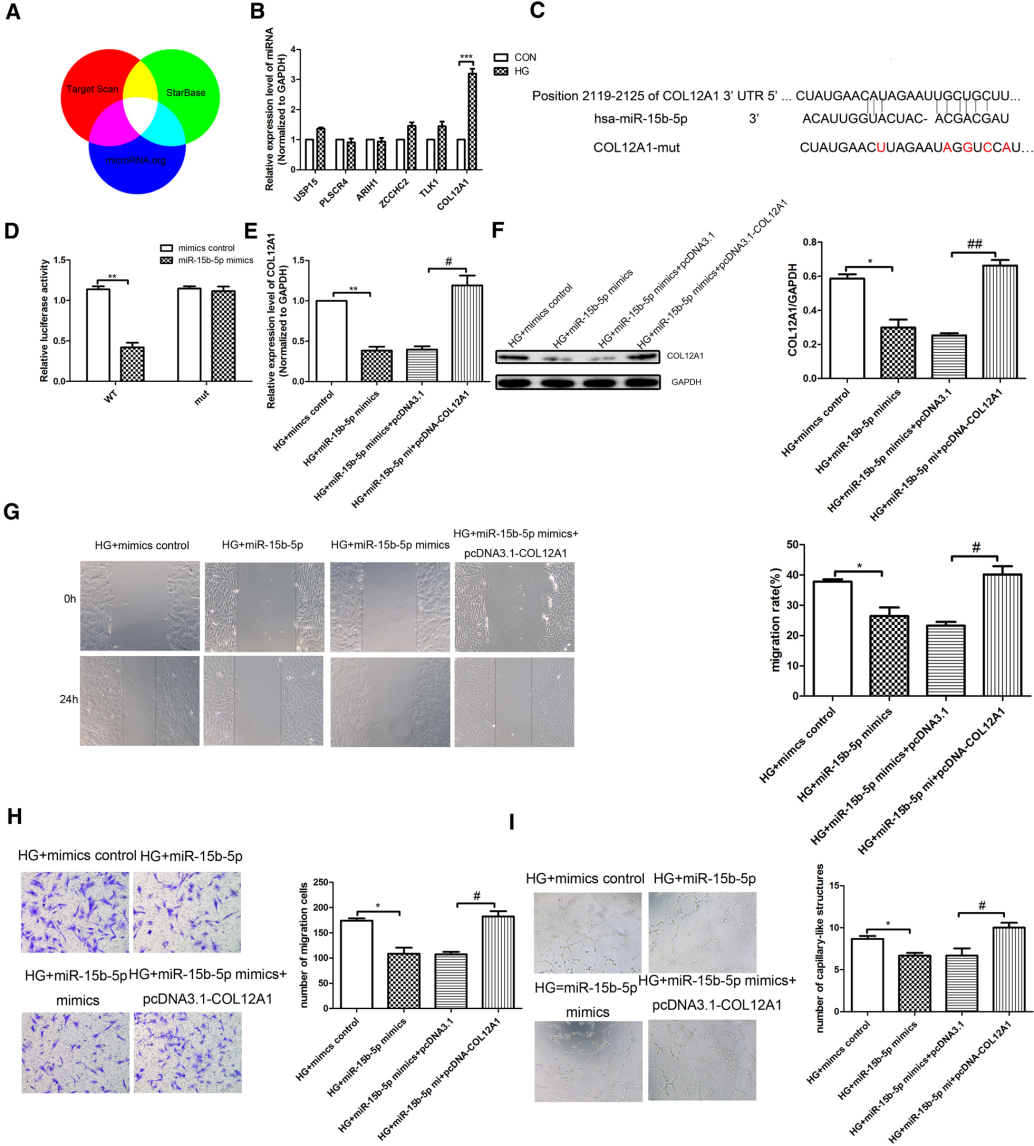
MiR-15b-5p regulates HRVECs function through targeting COL12A1. **A** A Veen diagram reveals the predicted target genes of miR-15b-5p from TargetScan, Star Base and microRNA.org. **B** For the 6 screened target genes, qRT-PCR was used to detect the expression difference induced by HG. **C** Luciferase reporter vectors containing the wild-type or mutant 3′ UTR of the COL12A1 were constructed according to the predicted binding sites of miR-15b-5p. **D** The histogram shows the changes of luciferase activities under different conditions. The HRVECs were co-transfected with luciferase reporter vector and miR-15b-5p mimics or mimics control. The expression of COL12A1 in HRVECs induced by HG was detected by qRT-PCR analysis (**E**) and western blot assay (**F**). **G** Wound healing assay was used to detect the invasion of HRVECs in different groups. **H** Transwell assay was used to detect the migration of HRVECs in different groups. **I** Tube formation experiment was performed to assess the tubule-forming ability of cells in different groups. The data was presented as mean ± SEM, n = 3 each group. Vs control/HG + mimics control group, *P < 0.05, **P < 0.01; vs HG + miR-15b-5p mimics + pcDNA3.1 group, ^#^P < 0.05, ^##^P < 0.01

Further experiments were performed to explore the effects of miR-15b-5p on HRVECs cell functions, such as cell invasion, migration and tube formation. First, according to Target Scan, Star base and microRNA.org, we predict the potential target genes of miR-15b-5p through bioinformatics analysis (Fig. 2A). QRT-PCR analysis was used to detect the difference of selected target genes. The results (Fig. 2B) showed that COL12A1 is the most significantly upregulated gene in HG-induced HRVECs. We further investigated whether the effects of miR-15b-5p on HRVECs were exerted through targeting COL12A1. Furthermore, TargetScan prediction and luciferase activity assay verified that miR-15b-5p had a binding relationship with COL12A1 (Fig. 2C). Luciferase activity assay results showed that a significant decrease in luciferase activity was observed in the group with the wild-type COL12A1 reporter (Luc-COL12A1-WT) plus miR-15b-5p mimics (Fig. 2D). In addition, we detected the expressions of COL12A1 in HG-induced HRVECs through qRT-PCR assay and western blot. As shown in Fig. 2E, F, we found that compared with HG + mimics control group, the mRNA and protein expressions of COL12A1 in HG + miR-15b-5p mimics group was reduced significantly. The expressions of COL12A1 in HG + miR-15b-5p mimics + pcDNA3.1-COL12A1 group was increased significantly compared with HG + miR-15b-5p mimics + pcDNA3.1 group. Moreover we evaluated the effects of miR-15b-5p in the HRVECs functions through targeting COL12A1. The wound healing results, transwell assay results and tube formation results (Fig. 2G–I) showed that compared with HG + mimics control group, the invasion, migration and tube formation of HRVECs was inhibited in HG + miR-15b-5p mimics group significantly. While the invasion, migration and tube formation of HRVECs in HG + miR-15b-5p mimics + pcDNA3.1-COL12A1 group was enhanced significantly compared with HG + miR-15b-5p mimics + pcDNA3.1 group. In summary, it revealed that miR-15b-5p affects the HRVEC function through targeting COL12A1.

### Circ_001209, a sponge of miR-15b-5p in HRVECs

**Fig. 3:**
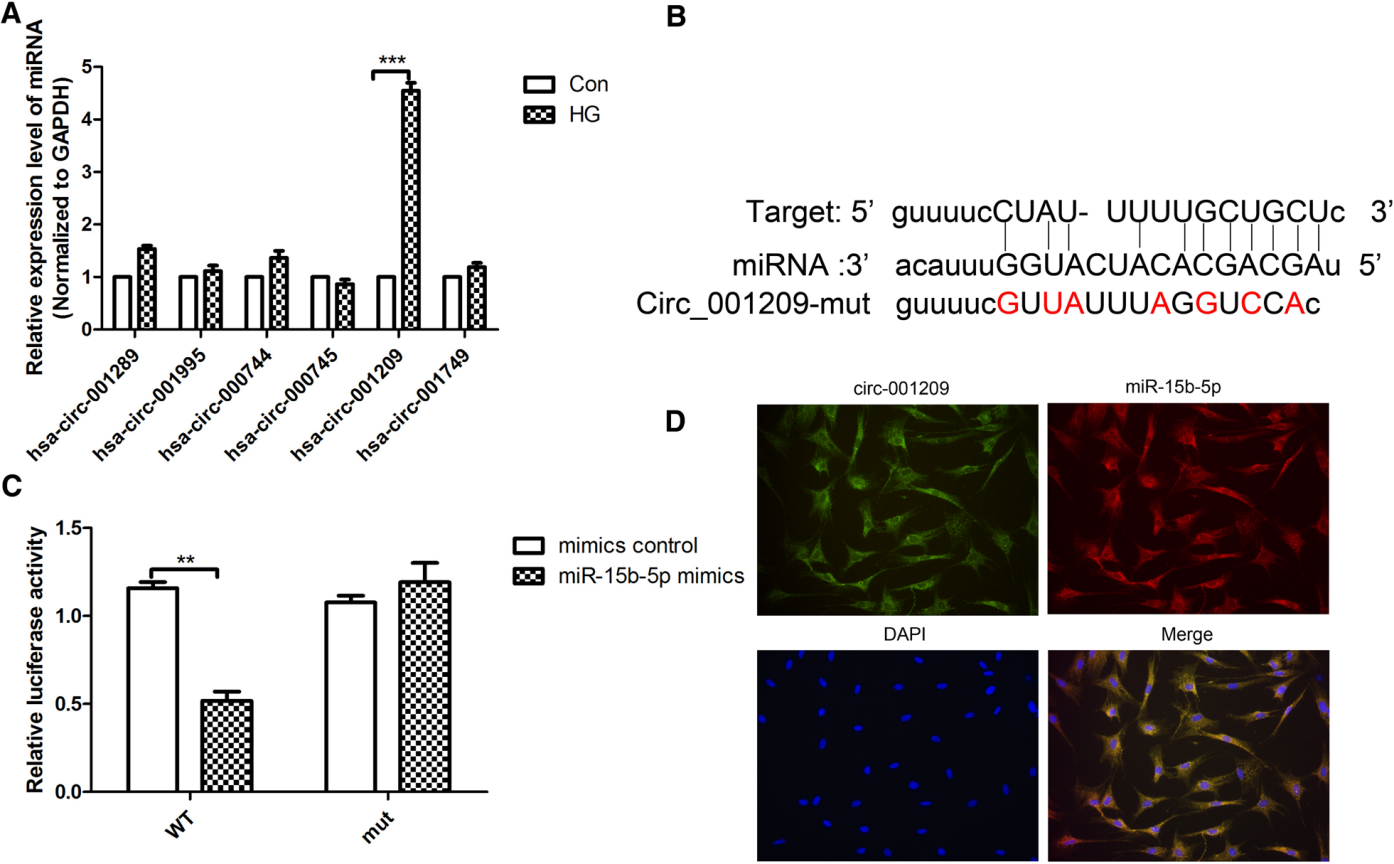
Circ-001209, a sponge of miR-15b-5p in HRVECs. **A** QRT-PCR analysis was performed to detect the mRNA expressions of different genes in HG induced HRVECs. **B** The predicted wild-type or mutated circ_001209 binding site in miR-15b-5p. **C** 293T cells were transfected with wide-type or mut circ_001209 reporter plasmid, and the luciferase reporter was performed to confirm the direct target sites. **D** RNA-FISH was utilized to examine the co-localization between circ_001209 (FITC-labelled) and miR-15b-5p (Cy3-labelled) in the cytoplasm of HRVECs cells. Nuclei were stained with DAPI. The data was presented as mean ± SEM, n = 3 each group. Vs con/mimics control group, *P < 0.05, **P < 0.01

CircRNAs, a novel type of non-coding RNAs, have been shown to play an important role in the pathological processes of some diseases through sponging miRNA. We screened circRNAs that might act as a sponge of miR-15b-5p using Targetscan, Starbase, and miRNA.org (Fig. 3A). The predictive results were overlapped with the microarray data, revealing differentially expressed circRNAs between control group and HG-induced HRVECs. Considering abundance and sequence length, the most promising candidates, circ_001209 was screened out through qRT-PCR analysis results (Fig. 3A). Based on the TargetScan prediction (Fig. 3B), we have verified that miR-15b-5p had a binding relationship with circ_001209. And dual luciferase activity results showed that miR-15b-5p mimic could significantly decrease luciferase activity (Fig. 3C). RNA-FISH experiment results showed that circ_001209 and miR-15b-5p were co-localized in the cytoplasm of HRMECs (Fig. 3D). The above results suggest that circ_001209 acts as a sponge of miR-15b-5p in HG-induced HRVECs.

### Circ_001209 regulates HRVECs function

**Fig. 4:**
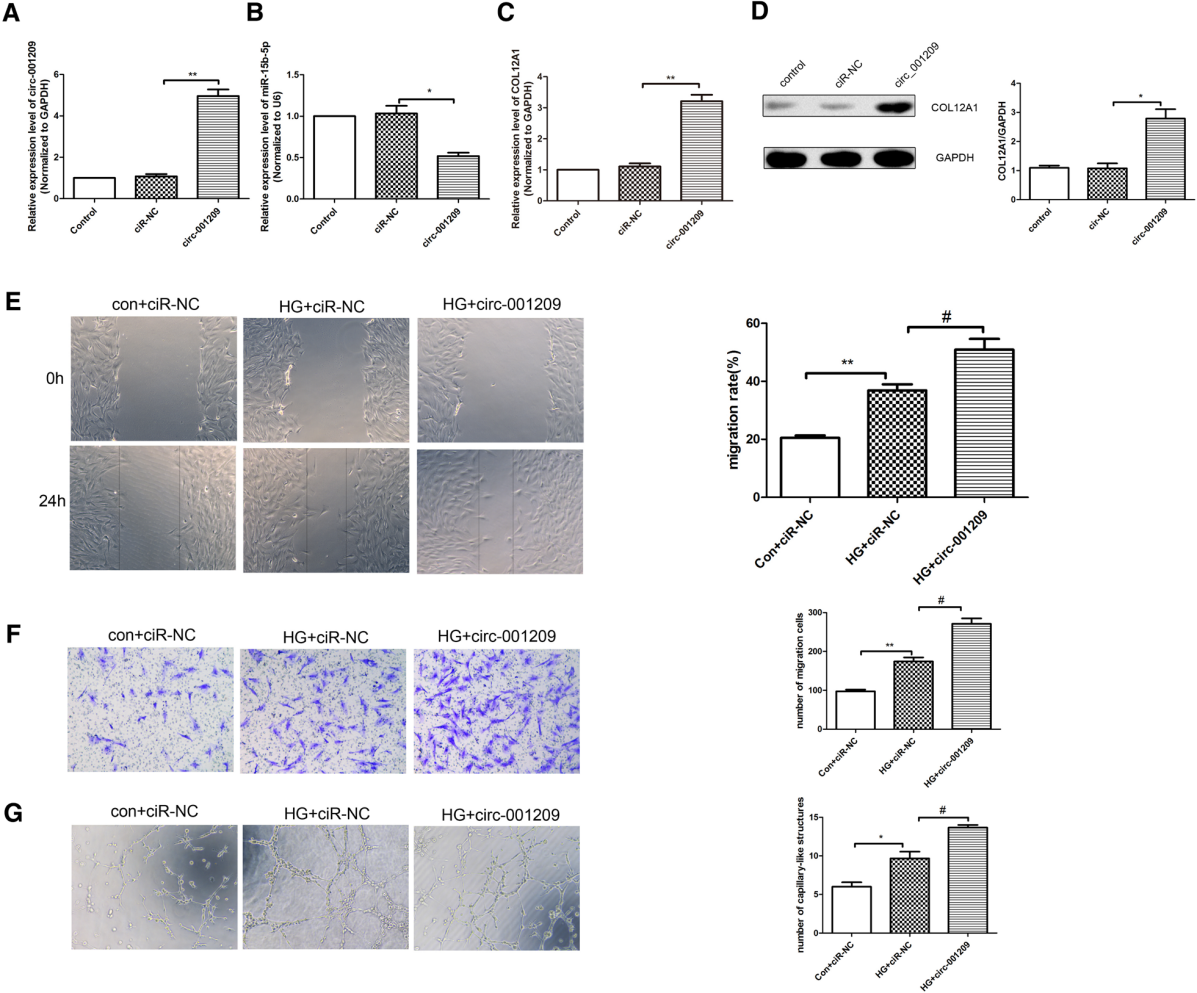
Circ_001209 regulates HRVECs function. QRT-PCR analysis was used to detect the expressions of circ_001209 (**A**), miR-15b-5p (**B**) and COL12A1 (**C**) in different groups. **D** Western blot assay was used to detect the protein expression of COL12A1 in different groups. **E** Wound healing assay was used to detect the invasion of HRVECs in different groups. **F** Transwell assay was used to detect the migration of HRVECs in different groups. **G** Tube formation experiment was performed to assess the tubule-forming ability of cells in different groups. The data was presented as mean ± SEM, n = 3 each group. Vs con + ciR-NC group, *P < 0.05, **P < 0.01; vs HG + ciR-NC group, ^#^P < 0.05, ^##^P < 0.01

To determine whether circ_001209 regulates HRVEC function by targeting miR-15b-5p, we used lentivirus containing circ_001209 sequence to stabilize the overexpression of circ_001209 in HRVECs. The results (Fig. 4A) showed that compared with ciR-NC group, the mRNA expression of circ_001209 in circ_001209 group was increased significantly. Furthermore, we also detected the changes of miR-15b-5p expression level in HRVECs through qRT-PCR analysis. As shown in Fig. 4B, it revealed that the expression of miR-15b-5p was downregulated significantly in circ_001209 group compared with ciR-NC group. QRT-PCR results (Fig. 4C) showed the mRNA expression of COL12A1 in circ_001209 was increased significantly compared with ciR-NC group. Consistent with qRT-PCR results, western blot results (Fig. 4D) also showed the same trend. Then, the wound healing results, transwell assay results and tube formation results (Fig. 4E–G) showed that compared with con + ciR-NC group, the invasion, migration and tube formation of HRMECs was enhanced in HG + ciR-NC group significantly. While the invasion, migration and tube formation of HRVECs in HG + circ_001209 group was enhanced significantly compared with HG + ciR-NC group. In summary, it revealed that circ_001209 could affect the HRVEC function.

### Circ_001209 regulates HRVEC function through targeting miR-15b-5p

**Fig. 5:**
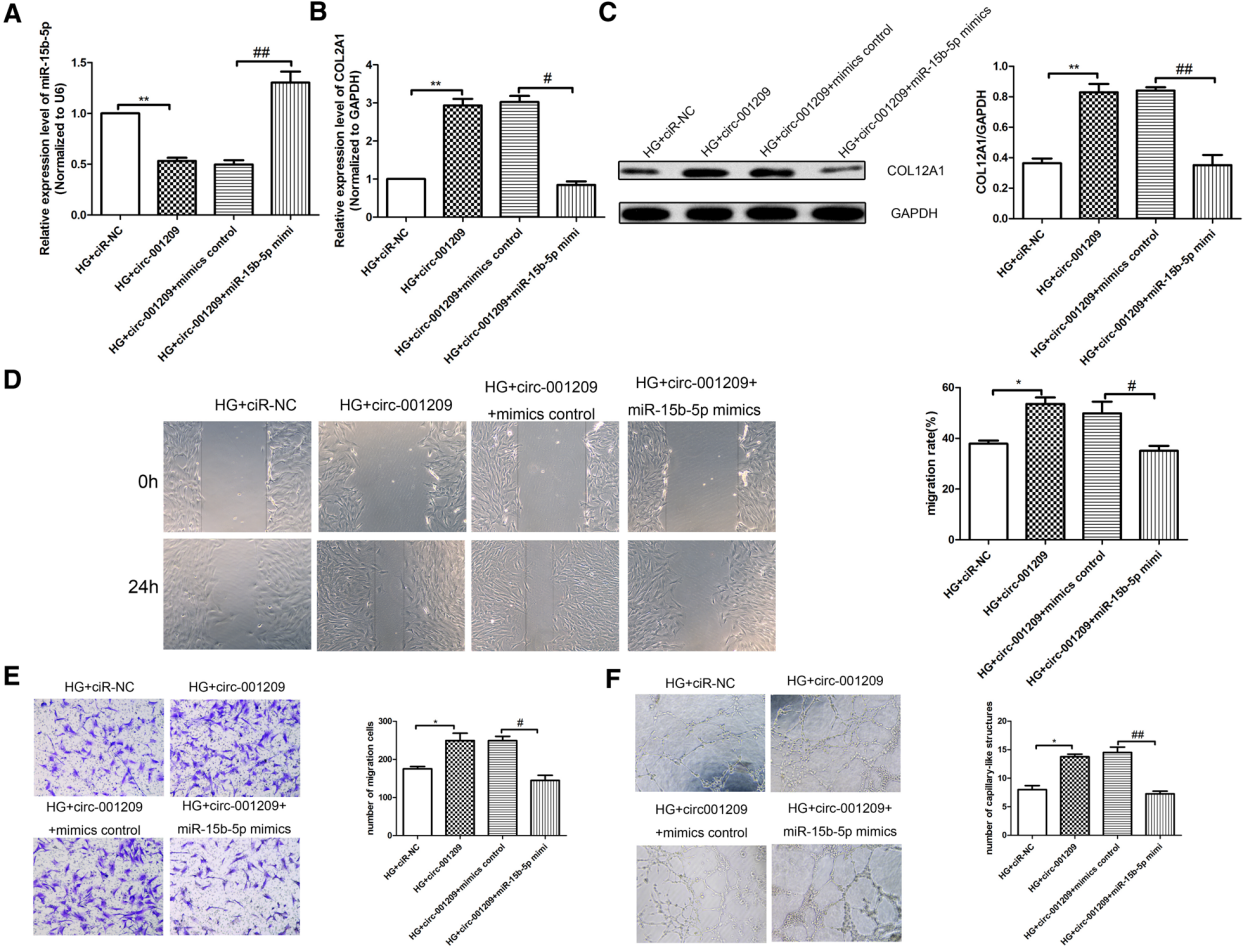
Circ_001209 regulates HRVEC function through targeting miR-15b-5p. **A** The relative expression of miR-15b-5p in HG-induced HRVECs was detected by qRT-PCR analysis. The expression level of COL12A1 in HG-induced HRVECs was detected by qRT-PCR analysis (**B**) and western blotting assay (**C**). **D** Wound healing assay was used to detect the invasion of HRVECs in different groups. **H** Transwell assay was used to detect the migration of HRVECs in different groups. **I** Tube formation experiment was performed to assess the tubule-forming ability of cells in different groups. **E** Transwell assay was used to detect the migration of HRVECs in different groups. **F** Tube formation experiment was performed to assess the tubule-forming ability of cells in different groups. The data was presented as mean ± SEM, n = 3 each group. Vs HG + ciR-NC group, *P < 0.05, **P < 0.01; vs HG + circ_001209 + mimics control group, ^#^P < 0.05, ^##^P < 0.01

To address whether circ_001209 regulates HRVEC function through targeting miR-15b-5p, lentivirus containing the circ_001209 sequence was used to stably overexpress circ_001209 in HRVECs. QRT-PCR results (Fig. 5A, B) showed that overexpression of circ_001209 significantly attenuated both the upregulation of miR-15b-5p and the downregulation of COL12A1 in HG induced HRVECs. Besides, western blot results (Fig. 5C) also showed the same trend of COL12A1 in HG-induced HRVECs. Wound healing assay, Transwell assay, and tube formation assays were used to evaluate the roles of circ_001209 under HG conditions in vitro. The results (Fig. 5D–F) showed that compared with HG + ciR-NC group, the invasion, migration and tube formation of HG induced HRVECs in HG + circ_001209 group was enhanced significantly. Compared with HG + circ_001209 + mimics control group, the invasion, migration and tube formation in HG + circ_001209 + miR-15b-5p mimics group was inhibited significantly. We found that the promoting effects of circ_001209 on invasion, migration, and tube formation in HRVECs were also attenuated by miR-15b-5p mimic transfection under HG conditions. The results suggested that circ_001209 regulates HRVECs function through targeting miR-15b-5p.

### Circ_001209 aggravates diabetes mellitus-induced retinal vascular dysfunction in vivo

**Fig. 6:**
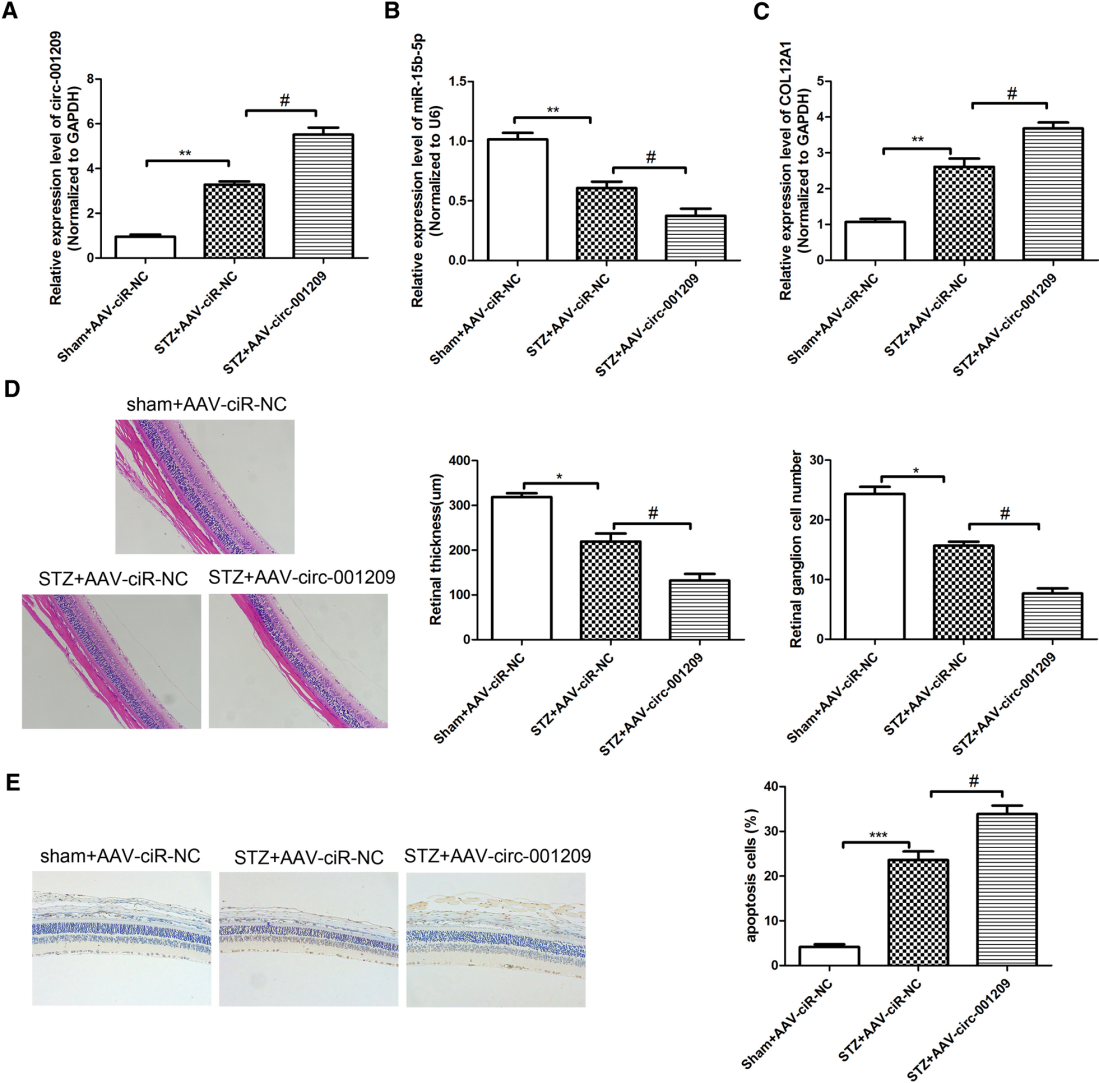
Circ_001209 aggravates diabetes mellitus-induced retinal vascular dysfunction in vivo. The retinal tissue was taken from the AAV-ciR-NC or AAV-circ_001209 injected diabetic rat at 2 months after injection. The expression of circ_001209 (**A**), miR-15b-5p (**B**) and COL12A1 (**C**) in the retinal tissue was detected by qRT-PCR analysis. Hematoxylin and eosin (H&E) staining was used to detect the pathological tissue damage induced by diabetic retinopathy in rats. **E** TUNEL staining images for retinal and the quantization diagram. The data are presented as mean ± SD. n = 6 each group. Vs sham ± AAV-ciR-NC group, *P  <  0.05, **P <  0.01; vs STZ + AAV-ciR-NC group, #P  <  0.05

We next investigated whether circ_001209 regulates retinal vascular dysfunction in vivo, further affecting visual function in diabetic rats. AAV-circ_001209 was injected intravitreally into diabetic rats. In diabetic retinas injected with AAV-ciR-NC, the level of circ_001209 was significantly increased compared with STZ + AAV-ciR-NC group. Then, the expression of circ_001209 was strongly increased in AAV-circ_001209-injected diabetic retinas compared with STZ + AAV-ciR-NC group (Fig. 6A). Similarly, qRT-PCR results (Fig. 6B) showed that the expression of miR-15b-5p in diabetic retinas was reduced significantly compared with sham group. Compared with STZ + AAV-ciR-NC group, the expression of miR-15b-5p was reduced significantly in STZ + AAV-circ_001209 group. We also detected the expression of COL12A1 in diabetic retinas. As shown in Fig. 6C, we found that compared with sham group, the expression of COL12A1 in diabetic retinas of STZ + AAV-ciR-NC group was significantly increased. The expression of COL12A1 in diabetic retinas of STZ + AAV-circ_001209 group was increased significantly compared with STZ + AAV-ciR-NC group. H&E staining was used to evaluate the pathological changes of retinopathy, as shown in Fig. 6D. It revealed that compared with sham group, retinal thickness was significantly thinner in diabetic rats. Compared with STZ + AAV-ciR-NC group, the retinal thickness was much thinner in STZ + AAV-circ_001209 group. TUNEL staining was performed to detect the apoptosis in diabetic retinal, the results (Fig. 6E) showed that the apoptosis in STZ + AAV-ciR-NC group was increased significantly compared with sham group. The apoptosis positive signal in STZ + AAV-circ_001209 group was significantly enhanced compared with STZ + AAV-ciR-NC group. The results further confirmed that circ_001209 could aggravate diabetes mellitus-induced retinal vascular dysfunction in vivo.

## Discussion

As a vascular complication in diabetes, DR could lead to blindness in the later stage, seriously affect the quality of life of patients, and also bring economic burden to families and society [20]. Although a large number of studies have explored the pathological mechanism of DR, there is still no effective clinical treatment strategy. Therefore, it is of great clinical significance to clarify the cellular mechanism of DR, develop new treatment strategies and improve the prognosis. The integrity of retinal vascular endothelial cells maintains stable retinal blood flow and ensures the supply of retinal blood oxygen and nutrients [21]. Hyperglycemia induces abnormal activation, proliferation, and migration of retinal vascular endothelial cells, which may lead to changes in retinal vascular function. There is increasing evidence [22] that hyperglycemia stimulates retinal vascular endothelial cell dysfunction as the pathological basis of DR.

In addition, previous studies [22] have shown that miRNAs can participate in the regulation of retinal vascular endothelial cell proliferation, activation, apoptosis and other physiological processes in the pathological state of diabetes, thus affecting the function of blood vessels. Zhu et al. [23] found that miR-20b-5p was upregualted markedly in HRVECs and patients with DR, which could promote the proliferation and exacerbate the progression of DR. Whereas, Zeng et al. [24] confirmed that miR-29b-3p promoted HRMECs apoptosis, which indicated that miR-29b-3p may be a key factor during the progression of DR. In our study, we found a potential correlation between the downregulation of miR-15b-5p and the dysfunction of retinal vascular endothelial cells. The expression of miR-15b-5p was significantly down-regulated in the HRVECs treated with HG and the retinal tissues of diabetic rats. Besides, the migration and proliferation of human retinal vascular endothelial cells promoted by HG could be significantly inhibited by miR-15b-5p mimics.

COL12A1, a gene encoding collagen type XII α 1 chain, is a typical molecule that regulates collagen expression and is involved in the formation of collagen in the cancer microenviroment [25]. Recently, some studies [26] have reported that COL12A1 was associated with the tumor invasion, migration and metastasis, the mechanism in DR has not been reported. In the further study of the regulation mechanism of miR-15b-5p on HRVECs, we found that among the 6 target genes (USP15, PLSCR4, ARIH1, ZCCHC2, TLK1, COL12A1) regulated by miR-15b-5p, the gene level of COL12A1 was the most significantly up-regulated in HG induced HRVECs. Besides, cellular functional experiments confirmed that miR-15b-5p directly inhibited the expression of COL12A1 and inhibited the proliferation, migration and angiogenesis of HRVECs induced by HG.

As a new type of non-coding RNA, circRNAs have closed-loop structure and are widely involved in cell proliferation, differentiation, apoptosis and other different cellular physiological or pathological processes [27]. Recent studies [28] have found that abnormal expression of circRNAs may be involved in pathological processes such as inflammation and vascular dysfunction in diabetic retinopathy by sponging microRNAs. Zou et al. [29] suggested that circRNA COL1A2 promoted angiogenesis by regulating the miR-29b/VEGF axis and aggravated the damage of diabetic retinopathy. Qin et al. [30] also found that circRNA-ENF532 plays a protective role in regulating vascular dysfunction in DR. In our study, combined with bioinformatics data analysis, we found that circ_001209 can directly target miR-15b-5p and regulate the expression of COL12A1 in HG induced HRVECs. Consistent with the prediction from bioinformatics, the dual-luciferase reporter assay demonstrated that miR-15b-5p binds circ_001209 at the target sites. RNA-FISH analysis showed co-localization of circ_001209 and miR-15b-5p in the HRVECs. These results demonstrated that circ_001209 functions as a sponge of miR-15b-5p, negatively regulating miR-15b-5p expression under diabetic conditions in retinal vascular tissue.

In the present study, the overexpression of circ_001209 aggravated the abnormal proliferation, migration and tube formation of HRVECs induced by HG. In addition, circ_001209 overexpression significantly increased the tubule-forming ability of retinal blood vessel cells induced by hyperglycemia. In addition, both H&E and TUNEL staining results in diabetic rats showed that circ_001209 overexpression led to abnormal pathological changes and dysfunction of retinal vessels. As for DR, the exact molecular mechanism of the non-coding RNA regulation still needs to be clarified through future research.

In conclusion, we have provided strong evidence that the upregulation of circ_001209 leads to retinal vascular dysfunction in diabetic retinopathy through the regulation of miR-15b-5p and COL12A1. Since circ_001209 could aggravate the pathological process of diabetic retinopathy, we could explore new therapeutic strategies for diabetic retinopathy in response to this mechanism of action, which will provide new ideas for clinical treatment of diabetic retinopathy.

## Data Availability

The data and materials for this study is available from the corresponding author on request if necessary.
